# Derivation and validation of a risk classification tree for patients with synovial sarcoma

**DOI:** 10.1002/cam4.4909

**Published:** 2022-06-07

**Authors:** Dylan V. Neel, Clement Ma, Natalie B. Collins, Jason L. Hornick, George D. Demetri, David S. Shulman

**Affiliations:** ^1^ Harvard Medical School Boston Massachusetts USA; ^2^ Centre for Addiction and Mental Health Toronto Ontario Canada; ^3^ Division of Biostatistics, Dalla Lana School of Public Health University of Toronto Toronto Ontario Canada; ^4^ Dana‐Farber/Boston Children's Cancer and Blood Disorders Center Harvard Medical School Boston Massachusetts USA; ^5^ Department of Pathology Brigham and Women's Hospital and Harvard Medical School Boston Massachusetts USA; ^6^ Dana‐Farber Cancer Institute and Ludwig Center at Harvard Boston Massachusetts USA

**Keywords:** oncology, prognostic factors, risk‐stratification, synovial sarcoma

## Abstract

**Background:**

Synovial sarcoma (SS) accounts for 8%–10% of all soft‐tissue sarcomas. Clinical presentation and outcomes vary, yet discrete risk groups based on validated prognostic indices are not defined for the full spectrum of patients with SS.

**Methods:**

We performed a retrospective cohort study using data from the SEER (surveillance, epidemiology, and end results program) database of SS patients who were <70 years of age at diagnosis. We constructed a recursive partitioning model of overall survival using a training cohort of 1063 patients with variables: Age at diagnosis, sex, race, ethnicity, primary site, tumor size, tumor grade, and stage. Based on this model, we grouped patients into three risk groups and estimated 5‐year overall survival for each group. We then applied these groups to a test cohort (*n* = 1063).

**Results:**

Our model identified three prognostic groups with significantly different overall survival: low risk (local/regional stage with either <21 years of age OR tumor <7.5 cm and female sex), intermediate‐risk (local/regional stage, age ≥ 21 years with either male sex and tumor <7.5 cm OR any sex with appendicular anatomic location) and high risk (local/regional stage, age ≥ 21 years, tumor size ≥7.5 cm and non‐appendicular location OR distant stage). Prognostic groups were applied to the test cohort, showing significantly different survival between groups (*p* < 0.0001).

**Conclusions:**

Our analysis yields an intuitive risk‐classification tree with discrete groups, which may provide useful information for researchers, patients, and clinicians. Prospective validation of this model may inform efforts at risk‐stratifying treatment.

## Graphical abstract

We used a multivariable recursive partitioning model to define prognostic groups for children and adults with synovial sarcoma. These risk groups provide clinicians and researchers with an intuitive framework for estimating individual patient risk.

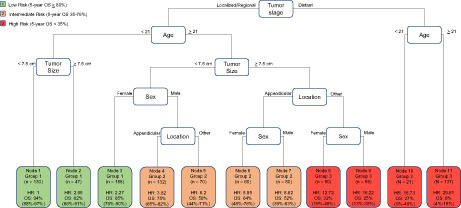

## 1 INTRODUCTION

Synovial sarcoma (SS) is an aggressive malignancy of mesenchymal origin and accounts for a significant proportion of soft tissue sarcomas (STS) seen in both children and young adults. The annual incidence is 2–3 cases per 100,000 people and typically presents between the ages of 15 and 30 years. The disease is genetically characterized by a *t*(*X*:18) translocation, fusing the *SS18* (formerly *SYT*) gene from chromosome 18 to either *SSX1*, *SSX2* or rarely *SSX4* from the X chromosome.^1^, ^2^ The SS18‐SSX fusion hijacks BAF remodeling complexes on chromatin to be redirected from selective enhancer sequences to broad polycomb domains. This aberrant localization drives a cancer‐supportive transcriptional program to induce the malignant transformation and progression to SS.^3^ While surgical excision is typically curative for patients with small localized tumors, cytotoxic chemotherapy, radiation, or multi‐kinase inhibition with pazopanib are generally accepted standard approaches to manage patients with unresectable, metastatic or recurrent disease.^4^ Currently, our ability to provide risk‐adjusted therapy to patients at initial presentation remains limited.

A prior analysis of SEER data from 1983 to 2005 for patients with SS demonstrated that children (<18 years) had superior outcomes to adults, and that the variables of female sex, extremity tumors, localized tumors, and tumors <5 cm were all associated with more favorable outcomes.^5^ Certain studies have shown that histologic grade, according to the French Fédération Nationale des Centres de Lutte Contre le Cancer (FNCLCC) tumor grade system, was prognostic.^6^, ^7^, ^8^ Several other studies have evaluated STS as a heterogeneous group in adults and non‐rhabdomyosarcoma soft tissue sarcomas (NRSTS) in children to determine prognostic factors.^9^, ^10^, ^11^ Tumor size, depth, site, histology and patient age were all found to influence risk of relapse and death. However, only a small minority of patients in these pooled analyses carried a diagnosis of SS. Pediatric patients (<21 years of age) have traditionally received stratified therapy based on tumor size and completeness of resection; patients with localized tumors <5 cm receive surgery alone, and patients with larger tumors or metastatic spread receive surgery, radiation therapy, and chemotherapy.^12^ A more recent report of 146 patients with SS treated on the COG study ARST0332 demonstrated that risk‐stratified treatment based on clinical features produced favorable outcomes in young patients with localized disease.^13^ Two studies have developed nomograms of patients with synovial sarcoma, however, these studies do not allow for contextual evaluation of risk factors, do they lend themselves to intuitive risk‐classification and included limited numbers of young patients.^14^, ^15^

Discrete risk groups have not been defined for the full spectrum of patients presenting with SS. Identification of discrete risk‐groups, especially to identify patients at higher risk of poor outcomes, is essential for the deployment of risk‐adapted therapy, which has led to improved outcomes for many cancer histologies. With multiple new therapeutic strategies on the horizon, identifying those patients who are unlikely to be cured with standard therapy is critical.

To address these gaps in SS risk‐stratification, we have analyzed data from the SEER database and utilized recursive partitioning to identify discrete prognostic groups. The database was parsed into training and test cohorts to build a classification and regression tree, define prognostic groups, and independently validate the designated groups.

## 2 MATERIALS AND METHODS

### 2.1 Patient population

We performed a retrospective cohort study of patients with SS (ICD‐O‐3: 9040/3, 9041/3, 9042/3, 9043/3) 70 years of age or younger with complete staging information. SEER diagnoses, whenever possible are based on pathologic diagnosis, as mentioned in the SEER Program Coding and Staging Manual 2018. Age 70 was used as a cut‐off given that treatment strategies and goals of care may differ significantly in much older patients with greater number of co‐morbidities. Life expectancy in the United States was between 70 and 80 years for the study period and in this dataset, death from non‐oncologic causes becomes a significant competing risk factor for patients above age 70. We utilized data from the SEER18 database for patients diagnosed during the years of 2000 to 2017 to define prognostic groups (data downloaded on June 1, 2020). Data from the SEER18 system were derived from 34.6% of the US population and provided data on cancer incidence, age at diagnosis, primary tumor site, tumor histology, grade/stage at diagnosis, and survival.^16^

### 2.2 Predictor variables

Clinical variables from the SEER database were abstracted in the following manner: age at diagnosis, sex (male, female), race (White, non‐White), primary site (appendicular, other), tumor size (cm), tumor histopathologic grade (well‐differentiated Grade I/moderately differentiated Grade II as “low‐grade”; and poorly differentiated grade III and undifferentiated anaplastic Grade IV as “high‐grade”) and stage (localized/regional and distant) (Table S1).^6^ Primary site location was divided into appendicular and “other” (CNS/leptomeningeal, head/neck, heart/mediastinum, lung/pleura, retroperitoneal, trunk, visceral), given the small number of cases (<1%) in certain subgroups such as CNS/leptomeningeal. In order to increase statistical power and parity between groups, we divided cases into appendicular (63%) and other (37%). For cases with very large tumor size (>30 cm), tumor size values were marked as missing, given concern for erroneous data collection (Table 1). Histologic subtype (monophasic, biphasic, not otherwise specified [NOS]) was reported in Table 1, but not included in the regression analysis given that it cannot be easily converted to a binary variable and histologic subtypes have not previously been demonstrated to be consistently prognostic.^6^ For continuous variables (age and tumor size), optimal cut‐points were obtained prior to model building as described in the analysis section below.

**TABLE 1:** Overall patient characteristics of 2126 patients from SEER database, and training and test cohorts

Characteristic	All SEER cases (*n* = 2126)	Training cohort (*n* = 1063)	Test Cohort (*n* = 1063)
Age at diagnosis (years)			
Median (range)	35 (<1–70)	34 (3–70)	37 (<1–70)
% ≥21 years of age	80.2	79.5	81.0
Sex (%)			
Male	53.5	52.7	54.3
Female	46.5	47.3	45.7
Race (%)			
White	80.1	79.5	80.7
Non‐White	19.9	20.5	19.3
Ethnicity (%)			
Hispanic	25.3	26.2	24.5
Not‐Hispanic	74.7	73.8	75.5
Tumor size^a^ (cm)			
Median (range)	6.5 (0.1–30.0)	6.2 (0.1–30.0)	6.6 (0.2–30.0)
% ≥7.5 cm	42.4	41.0	43.7
Stage (%)			
Local/Regional	84.4	85.1	83.6
Distant	15.6	14.9	16.4
Grade^b^ (%)			
Well differentiated Grade I	1.7	1.4	1.9
Moderately differentiated Grade II	22.6	22.9	22.4
Poorly differentiated Grade III	44.7	44.4	45.1
Undifferentiated anaplastic Grade IV	31.0	31.3	30.6
Histology (%)			
Monophasic	38.9	39.1	38.7
Biphasic	22.1	21.8	22.5
NOS	39.0	39.1	38.8
Primary location^c^ (%)			
Appendicular	63.2	63.0	63.3
Other	36.8	37.0	36.7
Year of diagnosis (%)			
Pre‐2010	45.5	46.8	44.2
2010 and beyond	54.5	53.2	55.8

^a^ Tumor size available for *n* = 1850.

^b^ Grade available for *n* = 1246.

^c^ Primary tumor location available for *n* = 2123.

### 2.3 Outcome variables

The primary clinical endpoint used in this study was overall survival (OS), which was defined as the time from diagnosis to the time of death due to any cause, with surviving patients censored at the time of last follow‐up.

### 2.4 Statistical analysis

Patient characteristics were summarized using descriptive statistics. Categorical variables were summarized using frequencies and proportions. Continuous variables were summarized using means, medians, and ranges.

Optimal cut‐point analysis for the continuous variables age and tumor size were performed using the R package “partykit” version 1.2.7.^17^ Relative to complexity parameter pruning methods (e.g., Rpart),^18^ conditional inference trees such as “partykit” reduce variable selection bias towards variables with more than a single cut‐point.^17^, ^19^ In this approach, the cut‐point most significantly associated with survival in the univariate fashion (smallest *p*‐value) was selected for branching and split using a goodness of split measure that optimizes between node separation using log‐rank statistics.^19^ The algorithm used a Bonferroni method to adjust for multiple testing at each split.

Once each variable was dichotomized, the classification tree was built using manual Cox proportion‐hazards regression models.^20^ Branch points were selected based on the variable at each level with the highest hazard for event and a *p*‐value <0.05. When there was no longer a statistically significant (*p* < 0.05) split, or a resultant node had fewer than 20 patients, the tree was stopped. For branch points of variables with missing data, cases without that data available were dropped from further analysis within that branch. Proportional hazards were checked for each model.

After defining terminal nodes, the 5‐year OS and 95% confidence interval for each derived node was estimated using Kaplan–Meier methods. The relative hazard ratio for death was calculated for each node in reference to the lowest risk group (Node 1).

Following creation of the survival tree using the training data, each node was assigned to a pre‐defined, clinically relevant, low‐risk (5‐year OS ≥80%), intermediate‐risk (5‐year OS 35%–79%), or high‐risk (5‐year OS <35%) group based on the 5‐year OS of the node. The exact cutoffs for these prognostic groups were assigned using clinical judgment, and closely resembled OS survival rates of assigned groups in similar types of analyses.^21^ Survival for each prognostic group in the training set was estimated using Kaplan–Meier methods and statistical significance determined using log‐rank tests.

We then applied these prognostic groups to the test cohort and used Kaplan–Meier methods to estimate 5‐year overall survival for each group. An overall log‐rank test was performed to determine whether the prognostic groups retained statistical significance in the test cohort.

The SEER database was accessed using SEER Stat version 7.0.5. Two‐sided *p*‐values <0.05 were considered statistically significant. Analyses were performed in R/RStudio version 4.0.0 and Stata 14.2.

## 3 RESULTS

### 3.1 Patient characteristics

**FIGURE 1:**
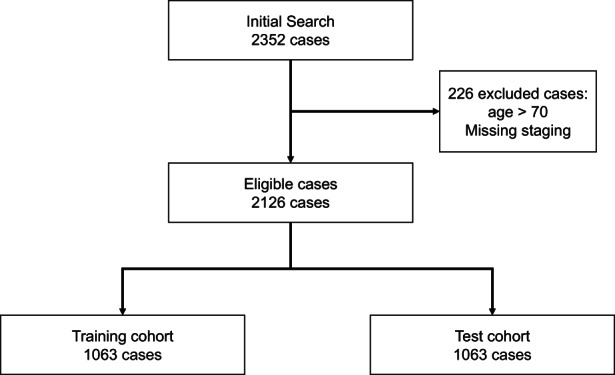
Consort diagram of SEER study population.

We identified 2352 cases in SEER18 with a histologic diagnosis of primary synovial sarcoma. We excluded 226 cases in which the age at diagnosis was greater than 70 or staging information was missing. The remaining 2126 patients were then randomly divided 1:1 into a training cohort (*n* = 1063) and a validation test cohort (*n* = 1063; Figure 1).

Patient characteristics of the training and test cohorts are summarized in Table 1. The median age of the overall cohort was 35 years (range: <1–70) with a slight male (53.5%) predominance. In the complete cohort, 15.6% of patients had metastatic disease at diagnosis and 63.2% of patients had appendicular tumors. Overall, there were more patients with monophasic tumors (38.9%) and histology categorized as NOS (39%) than patients with biphasic tumors (22.1%).

The 5‐year overall survival for the entire cohort was 62.1% (95% CI 59.8–64.3%) and did not differ significantly between the training and test groups (*p* = 0.45; Figure S1). The median follow‐up time for censored patients was 6.75 years (range: 1 month to 17.9 years).

For the purpose of utilizing clinically meaningful variables at the outset, we used regression analysis to identify the optimal cut‐points for age and tumor size in the training cohort. The cut‐point for age was determined to be 21 years, and the cut‐point for tumor size was 7.5 cm (Figure S2). For comparison, we compared a 7.5 cm cut‐point to a 5 cm cut‐point, given that 5 cm has traditionally been used as a cut‐point in soft tissue sarcoma. In our training cohort, the HR for event for a tumor size cut‐point of 5 cm was 3.70 (*p* < 0.001; 95% CI 2.78–4.92) compared to 3.55 (*p* < 0.001; 95% CI 2.84–4.45) for 7.5 cm. These variables were dichotomized for the entire cohort based on optimized cut‐points.

### 3.2 Defining risk groups in the training cohort

**FIGURE 2:**
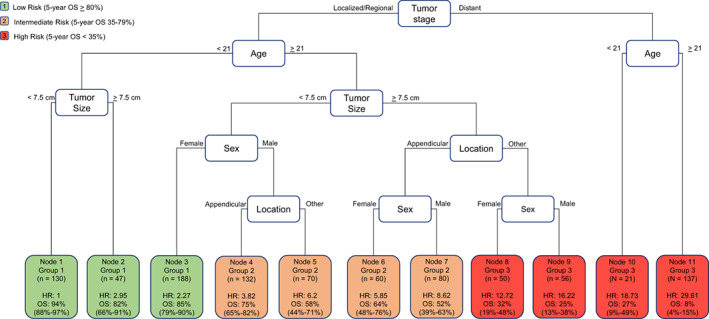
Classification and regression tree developed through recursive partitioning of the training cohort by overall survival (OS). Input variables included age at diagnosis, sex, race, ethnicity, primary location, stage, and tumor size. For each terminal node, hazard ratio relative to node 1 and 5‐year overall survival are included. Each node was placed in a low‐risk (green; 5‐year OS >80%), intermediate‐risk (orange; 5‐year OS 25%–79%) or high‐risk (red; 5‐year OS <35%) group based on pre‐specified 5‐year overall survival ranges.

Using the defined predictor variables, a classification tree with terminal nodes was generated using all patients in the training cohort (Figure 2). The strongest predictor of outcome was stage. Among patients with local/regional and metastatic tumors, age was the most important prognostic factor with older patients experiencing significantly worse outcomes. Among patients with local/regional disease, tumor size was the next most important risk factor. Sex and tumor location were significant only for patients with local/regional tumors, who were older. Among this group of patients who were over 21 years at diagnosis, we found that patients with tumors <7.5 cm who were female had excellent outcomes, and that patients who were over 21 years, had tumors ≥7.5 cm and a non‐appendicular primary site had dismal outcomes analogous to patients with metastatic disease. Survival estimates for each terminal node are shown in Figure 2.

**FIGURE 3:**
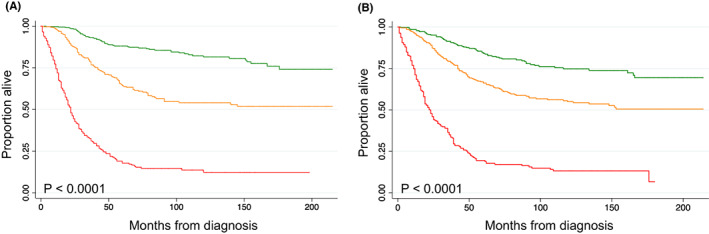
Estimated overall survival stratified by prognostic group for the (A) training (*p* < 0.0001) and (B) test cohorts (*p* < 0.0001).

We then grouped each terminal node into the pre‐defined low‐risk, intermediate‐risk, and high‐risk groups. The 5‐year overall survival estimates and characteristics for each prognostic group in the training cohort are shown in Table 2. We estimated survival for each resultant risk group (Figure 3A; *p* < 0.0001).

**TABLE 2:** Characteristics and outcomes of low‐, intermediate‐ and high‐risk groups

Prognostic group	Features	Train Cohort 5‐year OS (95% CI)	Test Cohort 5‐year OS (95% CI)
1 ‐ Low‐risk 5‐year OS ≥ 80%	Stage: Local/Regional Age: <21 Tumor size: Any (nodes 1 + 2)	88% (84–91)	84% (79–88)
	Stage: Local/Regional Age: ≥21 Tumor size: <7.5 cm Sex: Female (node 3)		
2 ‐ Intermediate‐risk 5‐year OS 35%–79%	Stage: Local/Regional Age: ≥21 Tumor size: < 7.5 cm Sex: Male Location: Any (nodes 4 + 5)	64% (58–70)	66% (60–71)
	Stage: Local/Regional Age: ≥21 Tumor size: ≥7.5 cm Location: Appendicular Sex: Any (nodes 6 + 7)		
3 ‐ High‐risk 5‐year OS < 35%	Stage: Local/Regional Age: ≥21 Tumor size: ≥7.5 cm Location: Non‐appendicular Sex: Any (nodes 8 + 9)	18% [13, 14, 15, 16, 17, 18, 19, 20, 21, 22, 23]	19% [14, 15, 16, 17, 18, 19, 20, 21, 22, 23, 24, 25]
	Stage: Distant Age: Any (node 10 + 11)		

### 3.3 Validation of risk group classification tree

Risk‐groups as defined in Figure 2 were then assigned to all patients in the test cohort. Kaplan–Meier curves of overall survival are shown in Figure 3B (*p* < 0.0001). We found that within the test cohort, patients in the low‐risk group had a 5‐year OS of 84% (95% CI 79–88%), whereas patients in the intermediate‐risk group had a 5‐year OS of 66% (95% CI 60–71%) and those in the high‐risk group had a 5‐year OS of 19% (95% CI 14–25%; Table 2).

## 4 DISCUSSION

This study utilized a regression analysis to generate a risk‐classification tree using training and test cohorts of patients from the SEER database. Such a regression analysis has the advantage over prior studies utilizing nomograms in that each clinical variable is evaluated within the context of specific subgroups of patients and provides an intuitive visual of outcomes.^14^, ^15^, ^22^, ^23^ Furthermore, not all of these studies included children and/or patients with metastatic disease. Prior prognostic tools used for soft tissue sarcomas, broadly defined, are not specific to SS, do not include children, and do not allow for variables to be assed in the context of specific patient subtypes.^24^, ^25^ For example, while having a large tumor is a poor prognostic factor, it is unknown whether this differs among patients who are young versus old. The first important finding from this analysis was that older patients with large non‐appendicular tumors had outcomes that were significantly worse than other patients with localized tumors and nearly as poor as patients with metastatic disease. Conversely, we demonstrate that while tumor size is an important clinical factor for patients with localized disease, young patients (<21 years) with localized disease demonstrated favorable survival outcomes regardless of tumor size at presentation.

Our analysis identified two groups of patients with an overall survival <35% at 5‐years, including patients with metastatic disease, and older patients with large primary tumors, presenting in non‐appendicular anatomic sites. These groups of patients warrant investigation of novel treatment strategies given the high‐risk of relapse. Among patients with metastatic disease, patients ≥21 years of age had particularly dismal outcomes; these older patients with metastatic disease had a five‐year OS of 8% (95% CI 4%–15%) compared to 27% (95% CI 9–49%) for patients <21 years of age at diagnosis with metastatic disease.

Young patients (≤ 18 years) with synovial sarcoma have been shown previously to demonstrate superior outcomes relative to patients who are older.^5^ Our analysis further validates this finding; interestingly, our regression analysis identified 21 years as the optimal cut‐point to stratify patients by age in this cohort. The reasons for this small age difference are not clear. In our analysis, age was a significant risk factor for patients with either small or large primary localized tumors. In prior analyses, younger patients appeared to have more favorable risk‐factors including smaller tumors that were more likely to be extremity primary tumors. However, younger patients, especially those <10 years of age, had better outcomes than adults when controlling for other variables.^5^ There is considerable speculation that this disparity has historically been due to more frequent use of chemotherapy in children when compared to adult patients, however, this is not known and was not possible to evaluate in our analysis. In our analysis, we found that female patients with localized tumors had a favorable prognosis relative to male counterparts. Whether biologic differences related to gender play a role in SS requires further clinical evaluation and maybe an interesting area of basic research. For example, it is interesting to speculate how random X‐inactivation of the translocated SSX segment may influence disease progression.^26^

Our study has several limitations. The SEER database provides an exceptional resource for the study of rare tumors such as SS, yet data were not complete for some variables utilized in our cohort (tumor size, grade, and primary location). Furthermore, tumor grade as recorded in SEER does not follow the FNCLCC tumor grading system which has been reported to be prognostic in other cohorts.^6^, ^7^, ^8^ Nevertheless, tumor size and primary location were available for most patients in the cohort. The SEER database does not require confirmation of fusion status in fusion‐positive tumors such as synovial sarcoma. However, in a recently published Phase 3 study from the COG of patients with synovial sarcoma, only one of 146 patients was excluded after central pathology review due to misdiagnosis.^13^ Additionally, SEER does not include details about treatment, which in particular may strongly influence clinical outcomes. Furthermore, study of the efficacy of systemic therapies in SS is required, and careful analyses of molecular genomic data may be helpful as well to inform risk‐stratification. At the present time, there are limited databases of this size that would have clinical data across the age spectrum with detailed treatment information. Lastly, though this study provides a validation cohort, our model warrants prospective validation.

In summary, our analysis provides a classification tree applicable to children, adolescents, and adults with SS. We identify a particularly high‐risk group of adult patients with large localized, non‐appendicular tumors, who may in part have poor outcomes given that local control can be more challenging in non‐appendicular sites than in appendicular sites. Conversely, we validate prior reports demonstrating more favorable outcomes for young patients, regardless of other clinical variables. While these findings provide an intuitive risk‐classification tree, this study supports the need for potentially more informative prognostic molecular markers in SS. This is particularly true given the recent major advances in the molecular mechanisms of oncogenesis in this fusion‐driven sarcoma with altered chromatin remodeling complexes.^3^, ^27^ Whereas patients with many other pediatric cancers including lymphoblastic lymphoma/leukemia and neuroblastoma receive risk‐stratified therapy that relies upon both clinical and molecular features, for patients with SS, like many other sarcomas, our treatment strategies continue to rely primarily on empiric clinical and pathologic features. Multiple new therapeutic strategies are on the horizon including, targeted immune‐based and engineered cell therapeutic strategies, and targeted protein degradation of molecular targets to induce synthetic lethality.^28^ Evaluation of such treatments in the setting of early‐stage, localized disease with the intent of increasing cure rates will require risk‐adapted clinical trials. Furthermore, studies should focus on the identification of molecular features to inform risk‐stratified therapy, as well as on improved therapeutic strategies beyond cytotoxic chemotherapy such as therapies. Evaluation of molecular markers in the context a regression model such as that presented here, may provide a more robust risk‐stratification model fit for clinical trial adaptation.

## AUTHOR CONTRIBUTIONS

Conceptualization: David S. Shulman. Study design and methodology: David S. Shulman, Dylan V. Neeland Clement Ma. Statistical analysis: David S. Shulmanand Dylan V. Neel. Writing‐original draft: David S. Shulmanand Dylan V. Neel. Writing‐review & editing – Clement Ma, George D. Demetri, Jason L. Hornick, Natalie B. Collins.

## FUNDING INFORMATION

This work was supported by Alex's Lemonade Stand Foundation (DSS) and the Harvard Catalyst KL2/CMeRIT program 5KL2TR002542 (DSS). The project described was also supported by award number T32GM007753 from the National Institute of General Medical Sciences (DVN). The content is solely the responsibility of the authors and does not necessarily represent the official views of the National Institute of General Medical Sciences or the National Institutes of Health.

## CONFLICT OF INTEREST

GDD: Scientific consultant with sponsored research to Dana‐Farber: Bayer, Pfizer, Novartis, Epizyme, Roche/Genentech, Epizyme, LOXO Oncology, AbbVie, GlaxoSmithKline, Janssen, PharmaMar, Daiichi‐Sankyo, AdaptImmune. Scientific consultant: GlaxoSmithKline, EMD‐Serono, Sanofi, ICON plc, MEDSCAPE, Mirati, Synlogic, WCG/Arsenal Capital, MJ Hennessey/OncLive, C4 Therapeutics, McCann Health, Rain Therapeutics. Consultant/SAB member with minor equity holding: G1 Therapeutics, Caris Life Sciences, Erasca Pharmaceuticals, RELAY Therapeutics, Bessor Pharmaceuticals, Champions Biotechnology, CellCarta, ikena Oncology, Kojin Therapeutics. Board of Directors member and Scientific Advisory Board Consultant with minor equity holding: Blueprint Medicines. Patents/Royalties: Novartis royalty to Dana‐Farber for “use patent” of imatinib in GIST. Non‐Financial Interests: AACR Science Policy and Government Affairs Committee Chair, Alexandria Real Estate Equities.

## ETHICAL APPROVAL STATEMENT

Ethical approval and informed consent for human subjects were not necessary for this study, as it was conducted on publicly available de‐identified data (SEER).

## Supporting information


Table S1
Figure S1
Figure S2

## Data Availability

Datasets generated during this study are presented in the figures, available for download from SEER, and available from the corresponding author upon reasonable.
