# Supplementary material for: Derivation and validation of a risk classification tree for patients with synovial sarcoma
Source: Cancer Med. 2022 Jun 7;12(1):170–8. doi: 10.1002/cam4.4909 (PMC9844650; doi:10.1002/cam4.4909)
Supplement: Supplementary file 1 — Table S1 Figure S1 Figure S2 [file CAM4-12-170-s001.docx]

**SUPPLEMENTAL MATERIALS**

| **Supplementary Table 1**: Response and predictor variables in the SEER database. | | |
| --- | --- | --- |
| **Predictor Variable** | **SEER nomenclature** | **Variable coding** |
| **Survival** | Survival months | Continuous (months) |
| **Event outcome** | Vital Status Recode | 0 = Censor  1 = Death |
| **Race** | Race | White  Non-White |
| **Ethnicity** | Ethnicity | Hispanic  Non-Hispanic |
| **Age** | Age at diagnosis | Continuous (yrs)  (Binarized 21 years cutpoint) |
| **Sex** | Sex | Male  Female |
| **Tumor size** | TS Summary 2016  CS Tumor Size 2004-2015  EOD 10 | Continuous (mm)  Binarized (7.5 cm cutpoint) |
| **Stage** | Stage A  Summary Stage 2000 (1998+)  SEER combined summary stage 2000 | Local  Regional  Distant |
| **Grade** | Grade | I, II (low grade)  III, IV (high grade) |
| **Histology** | Histology Recode ICD-O-3/WHO 2008 | 9040/3 (NOS)  9041/3 (Spindle Cell, monophasic)  9042/3 (Epithelioid, monophasic)  9043/3 (biphasic) |
| **Primary location** | Primary Site – labeled | Appendicular  Other |

**Supplementary Figure 1:** Overall survival curves for training (n=1063) and test (n=1063) cohorts of synovial sarcoma patients, truncated at 200 months of follow-up (p=0.45).

**Supplementary Figure 2:** Binarization of age at diagnosis and tumor size variables using recursive partitioning (partykit). Age and tumor size were individually analyzed and the single split with the most significant p-value was selected as the optimal cut-point. These cut-points were used to create binary variables and used in the final decision tree model with other independent variables.
